# Improved ECG Watermarking Technique Using Curvelet Transform

**DOI:** 10.3390/s20102941

**Published:** 2020-05-22

**Authors:** Lalit Mohan Goyal, Mamta Mittal, Ranjeeta Kaushik, Amit Verma, Iqbaldeep Kaur, Sudipta Roy, Tai-hoon Kim

**Affiliations:** 1 Department of Computer Engineering, J.C. Bose University of Sc. & Technology, YMCA, Faridaba 121006, India; lalitgoyal78@gmail.com; 2 Department of Computer Science & Engineering, G.B. Pant Govt. Engineering College, New Delhi 110020, India; mittalmamta@gbpec.edu.in; 3 Department of Computer Science & Engineering, Chandigarh Group of Colleges, Mohali 140307, India; ranjeetacec2012@gmail.com (R.K.); dramitverma.cu@gmail.com (A.V.); eriqbaldeepkaur@gmail.com (I.K.); 4 PRTTL, Washington University in Saint Louis, Saint Louis, MO 63110, USA; sudiptaroy@wustl.edu; 5 Computer Sc. Department, School of Economics and Management, Beijing Jiaotong University, Beijing 100044, China

**Keywords:** ECG, steganography, curvelet transform, clustering, performance metric

## Abstract

Hiding data in electrocardiogram signals are a big challenge due to the embedded information that can hamper the accuracy of disease detection. On the other hand, hiding data into ECG signals provides more security for, and authenticity of, the patient’s data. Some recent studies used non-blind watermarking techniques to embed patient information and data of a patient into ECG signals. However, these techniques are not robust against attacks with noise and show a low performance in terms of parameters such as peak signal to noise ratio (PSNR), normalized correlation (NC), mean square error (MSE), percentage residual difference (PRD), bit error rate (BER), structure similarity index measure (SSIM). In this study, an improved blind ECG-watermarking technique is proposed to embed the information of the patient’s data into the ECG signals using curvelet transform. The Euclidean distance between every two curvelet coefficients was computed to cluster the curvelet coefficients and after this, data were embedded into the selected clusters. This was an improvement not only in terms of extracting a hidden message from the watermarked ECG signals, but also robust against image-processing attacks. Performance metrics of SSIM, NC, PSNR and BER were used to measure the superiority of presented work. KL divergence and PRD were also used to reveal data hiding in curvelet coefficients of ECG without disturbing the original signal. The simulation results also demonstrated that the clustering method in the curvelet domain provided the best performance—even when the hidden messages were large size.

## 1. Introduction

The health of a patient can be constantly be monitored with the help of medical devices and digital communication. The old patients can send their physiological signals to the hospitals to avoid a repeated visit to the hospital. However, this technology has some concern about the security and content of the information. If the patients broadcast their medical or private information on the Internet in that case security is an important issue. Information broadcast without security can be assessed, modified, hacked by some unauthorized user [1]. The privacy of the patient can be leaked and affects the ability to diagnose due to unauthorized access. United States was passed an Act in 1996 as the Health Insurance Portability and Accountability Act (HIPAA) to protect patient privacy (personal data). This act was instructed about security regarding the communicating and storing of confidential and private information [2].

One possible solution to secure and protect ECG information is using cryptography, in which the original signal is encrypted at the sender side and decrypted at the receiver end [3]. However, the protection is not robust against data piracy as well as changes the bio-medical information that hampers the ability to diagnose. In that sense, steganography and watermarking are the alternative approaches to secure the patient’s data and information. A steganography approach [4,5] provides the worthy embedding capacity, even though it preserves the invisibility of embedded data. However, the hidden information would fail to extract if marked signals were corrupted during the transmission. Thus, the watermark needs to resist against geometric operations like rotation, noise addition and cropping [6]. However limited embedded information size of the watermark can affect the imperceptibility of the watermarked signal.

Nowadays, few authors have considered some method to hide patients’ information as a piece of copyright information in electrocardiogram (ECG) signals, magnetic resonance imaging (MRI) and electroencephalogram (EEG) [7,8]. The security of ECG signals using watermarking is still a developing area. Embedding a copyright message in an ECG signal is a sophisticated task, as the ability to diagnose the disease is dependent on the T and P waves of the QRS. Here QRS is the name for the combination of three of the graphical deflections seen on a typical electrocardiogram (EKG or ECG). Hence, the effects of data on T and P wave reduction are essential to cover the data in the ECG signal. Two watermarking approaches are used to embed a watermark in ECG signals. One is a spatial domain that modifies pixel intensities, and another one is the frequency domain, in which frequency is modified. In the spatial domain, a hidden message is embedded in the time domain of the original signal. A reversible ECG steganography method that is based on the coefficients of alignment and is resistant to image-processing attacks has been proposed by Yang et al. [9]. Some authors have used discrete wavelets transform (DWT) to embed a watermark. Zhang et al. [10] presented a reversible ECG steganography using DWT. Tseng et al. [11] also used the wavelet transform and compression for hiding the copyright information into ECG signals. The combination of singular value decomposition (SVD) and DWT for hiding data in ECG signals has presented by Jero et al. [12]. However, the wavelet transform is not able to represent the curvelet.

The author [12,13] also examined that the copyright information inserted in the High-High (HH) band offers the finest imperceptibility in DWT. However, the wavelet transforms not efficiently represent the curved lines in that existing DWT method due to the two-directional parameters such as scale and translation. Thus, some new curvelet transform is required to characterize 2-D or 3-D singularity. This transform having three parameters such as scale, translation and orientation. The three parameters are easy to represent curved lines. ECG signals are a mixture of curved lines and peak values. The curved lines play a very important role in diagnosing disease. The Shearlet transform is also an alternative to choose as an embedding approach. The Shearlet transform can deliver optimally sparse approximations for cartoon-like functions. This transform function was also used to hide the data.

Some researchers [14,15,16,17] also utilize the curvelet transform in ECG steganography. They hide the data into ECG by manipulating the curvelet coefficients. Jero et al. [15] represent an ECG steganography technique by using the n × n sequence method and modified the curvelet coefficients by an adaptive technique. A novel technique for ECG steganography using quantization has proposed by Jero et al. [16]. In this technique, the coefficients were selected based on the values equal to zero or close to zero. Patil et al. [17] proposed the ECG-watermarking using a curvelet by evaluating the coefficients and embed binary information into ECG signals with the help of property of quantization and least significant bit (LSB). Thanki [18] analyzed the hybrid multi-biometric technique by exploiting the fast discrete curvelet transform.

In this study, [18], the authors have investigated a technique of ECG-watermarking (using watermarking with compressive sensing theory) that hides the patient’s information into the curvelet domain of ECG. To maintain the diagnosis process and the QRS complex, P and T wave attributes, the curvelet coefficient clustering approach is used. For the demonstration, the MIT-BIH arrhythmia database is used to collect the ECG signals [19]. The performance of the proposed technique has been measured by the PSNR, NC, KL, PRD, SSIM, MSE, and BER parameters to show the imperceptibility of the watermarked ECG signal. The clustering approach in curvelet coefficients provides the invisibility and robustness of embedded watermark as well as provides the good quality of extracted watermark even from image-processing attacks. The performance of the proposed technique considering different sizes of the watermark and different selection of clusters has also been presented.

## 2. Curvelet Transform

Mainly the former transform, which is the ridgelet transform represents lines and curves in a very informative manner. The ridgelet transform solitary works for line singularities. There are inadequate directional features in ridgelet transform and not follows the scaling law. However, curvelet transforms added a new parameter called orientation that gives more information about the angles [20,21,22]. The basic unit of the curvelet is the ridgelet. Though the curvelet has higher directional representations than other multiresolution representations such as wavelets, ridgelet and random. The ccurvelet transform usages the scaling law in its structure.

Candes and Donoho [20] presented a new transform called the curvelet transform. For the curvelet transform, the first ridgelet transform is needed on each wedge. Ridgelet computation is a very costly procedure and the computation cost of curvelet is slow [23,24,25]. The ridgelet on each wedge is discarded to make the curvelet faster computation.

Curvelet transform is the function with three parameters: orientation, scale and translation as discussed, so assume ϑ(s,o,t) to be the function plane as defined in below equation:

(1) $$T_{\;\vartheta }=\overset{1}{\underset{R^{2}}{\int }}f\left(\alpha \right)\bar{\phi_{\;\vartheta }\left(\alpha \right)}\;\mathrm{dx}=\int \hat{f}\left(u\right)W_{s}\left(Q_{o}u\right)e^{i<\alpha_{t}^{o},u>}\;*\;\frac{1}{{\left(2\pi \right)}^{2}}$$

where s = scale parameter s = 0,1…, o = orientation 0,1,2…, t = translation where $t=\left(t_{1,}t_{2,}\right);{\;t}_{1,},t_{2,}\;\in Z$, $Q_{o\;}$ = rotation by o radian, $W_{s}$ = polar wedge window. Define coarse scale curvelet in below equations.

(2) $$\phi_{j,0,k\;}=\phi_{j,0}\left(x-2^{-j,0,k}\right)$$

(3) $${\hat{\phi }}_{j,o}\left(w\right)=2^{-j0}A_{0}\left(2^{-j,0}\left|w\right|\right)$$

The continuous-time window smoothly extracts the frequency near the dyadic corona and near the angle. In digital curvelet transform, a Cartesian corona is based on concentric square and shears instead of circles.

A new transform—the contourlets transform—is also a multiresolution directional transform, as it proficiently imprecise images prepared of smooth regions disconnected by smooth boundaries. It encouraged by the Human Visual System (HVS). The difference between two is that the curvelet transform was developed in a continuous domain, whereas the contourlet transform was planned as a directional multiresolution transform in a discrete domain. The curvelet transform can capture the smoothness of the curve of images with different elongated shapes and in variety of directions.

## 3. Proposed Method

In this application, the watermark is embedded into the ECG signal, as these signals contains an enormous number of curved surfaces. The curvelet transform authoritatively decomposes an image at different angles and the scales it to represent more curvilinear objects and have better edge representations. Hence, there is motivation to embed watermark on ECG signal by using curvelet transform without affecting the ability to diagnose.

### 3.1. Conversion of the ECG Signal into an Image

**Figure 1:**
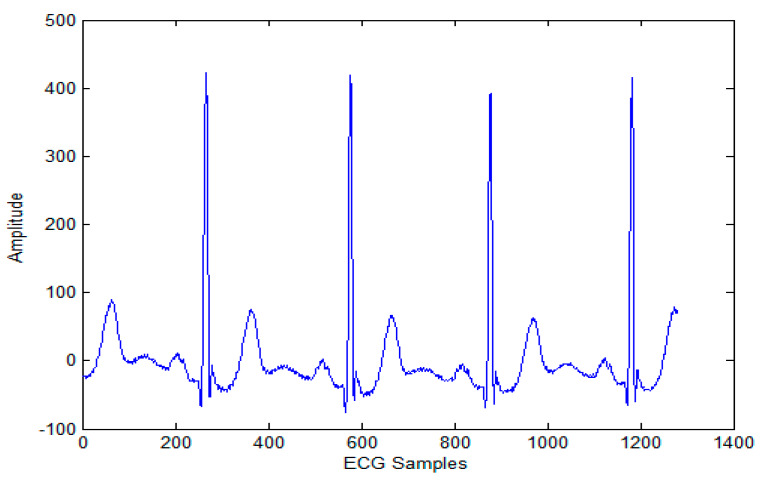
Original 1-D electrocardiogram.

**Figure 2:**
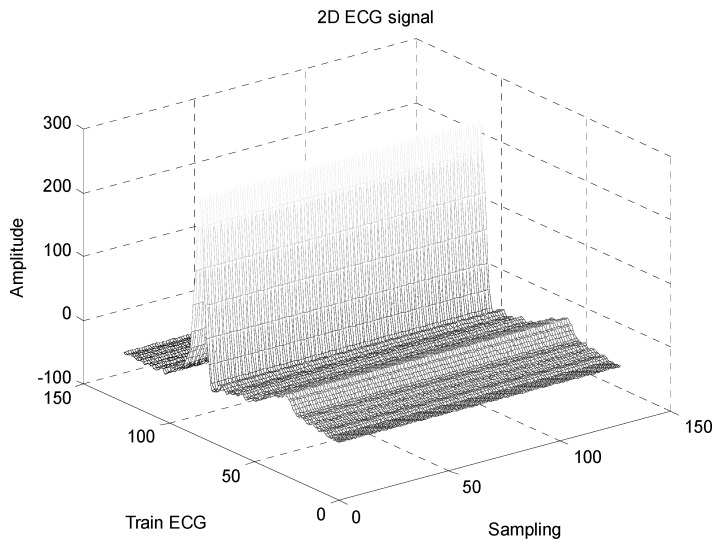
2-DECG image.

The biomedical ECG 1-D signal from the MIT-BIH arrhythmia database [19] sampled at 128 Hz was in this study. Figure 1 shows the 1D-ECG signal. P waves and T waves in the ECG QRS complex are the fundamental attributes for diagnosing any disease. The Tompkins algorithm [26] is used to identify P waves and T waves in ECG. In this, band-pass filters are used to filter the ECG signal. The signals are squared, and these squared signals are averaged by the window function to exclude noise, hence forming the ECG signal. The fiducial mask is attained by focusing the QRS attributes on a distinct instantaneous time. Then, its explorations back for missed QRS complexes and removes the duplicate detections. Lastly, the R-wave and QRS attributes are identified. P waves and T are utilized to alter the 1D-ECG signal into a 2-D image. Sampling rate 128 Hz is used and for each ECG trained signal fiducial points are calculated. From each calculated fiducial mask 64 points are utilized [12]. Now the ECG signal is changed into the image. The size of the 2-D image depends upon the number of ECG trains. There are some data losses due to the selected arguments employed to transform the 2D-ECG image into a 1D-ECG signal. However, this data loss is negligible and does not affect the ability to diagnose of the disease. The 2D-ECG image produced from Figure 1 is shown in Figure 2.

### 3.2. Transform ECG Image into Curvelet Transform

A fast discrete curvelet transform (FDCT) is applied to transform the host ECG image into the frequency domain. After applying FDCT, we obtained the number of scales k scale = log_2_ k − 2, where k × k is the dimensions of the ECG image. Subsequently, the decomposition Coarse, fine and detail level of coefficients are obtained. From these three levels, we can embed our data at any level. The invisibility and robustness can be decided from the selection of the level. In a coarse-level maintain most of the energy of image to give more invisibility.

### 3.3. Processing Patient Information

**Figure 3:**
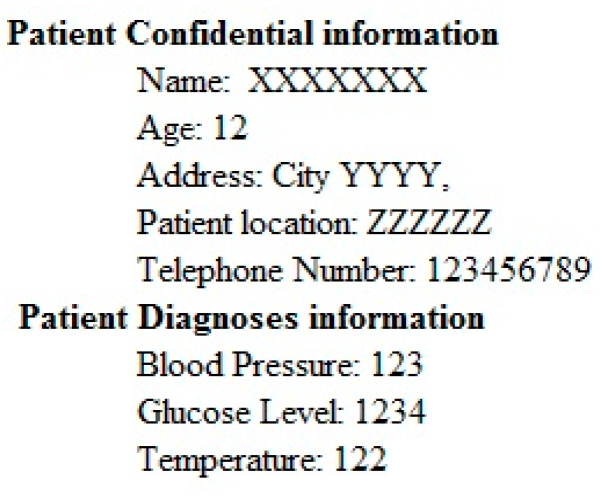
Image of patient information.

The main aim of this step is to gather the information of patients and development the patient information that non-authorized entity should not get admittance or tamper the personal evidence of the patient. Private information is gathered in the form of a text file then the text file is transformed into the binary image. The implanting the information in the ECG is easygoing and offers a good quality of mined watermark. Sample of patient information that could be implanted in ECG signals by modified the coefficients of curvelet transform as shown in Figure 3.

### 3.4. Curvelet Coefficients Clustering

In the curvelet coefficients selected the embedding domain is coarse-level (D) comprises K number. With the help of Euclidean distance, D is divided into m number of subclasses. Non-similarity measure between the curvelet coefficients and the cluster center is used to find Euclidean distance. Let P is the maximum coefficient and p is the minimum coefficient in the D. If p > 0 then we take the supreme coefficient P. Then preliminary cluster centers are selected after that the coarse-level D decomposed into B equal intervals. Then find out cluster’s center by using the following equations:

(4) $$c_{0}=0,\;c_{i}=\frac{i}{X}\;P,\;c\;=\frac{i}{X}\;p$$

where 1≤i≤X −1. For each cluster, calculate the Euclidean distance using ${\mathrm{ED}}_{i,j}=|{\;D}_{i}-D_{j}|$. After that categorize the coefficients rendering to the smallest Euclidean distance and placed the coefficient equivalent to the group center. The entire number of clusters is 2X − 1. They are R_i_, R_0_, R_-i_. Formerly apprise the cluster’s center by spending a threshold, i.e., (T = 0.1), V_j_ = average value of R_i_, Y_i_ = V_i_ − V_j_, if $Y_{i}>T$ then update the group epicenter. This method distributes coefficients into groups ranging from R_(X−1)_ to R_−(X−1)._ Patient information is implanted to coefficients of groups among R(_X−1_) − R_0_ and R_0_ − R_−(X−1)_.

### 3.5. Watermark Embedding

Select the cluster where do you wants to hide the patient’s information. Let, it be group R_i_ to R_j_. Change name the particular cluster G_1_, G_2_... G_k_, where k = 2(I − j) + 2. For each cluster G_i_, the radius of the groups are ${\mathrm{radi}}_{i1}=c_{i}-\max\left(G_{i}\right)$, ${\mathrm{radi}}_{i2}=c_{i}-min(G_{i}$).

Where, ${\mathrm{radi}}_{i1}$ and ${\mathrm{radi}}_{i2}$ are the radius of cluster G_i_ and c_i_ = center of the G_i._ For, respectively carefully chosen cluster q calculate following:

(5) l0(q)= ci−aA∗radii1

(6) l1(q)=ci−(a−1)A∗radii1

(7) L0(q)= ci+aA∗radii2

(8) L1(q)=ci+(a−1)A∗radii2

where A = numeral no. X = grouping for amount of cluster classes. a=1,2,……A.

Each coefficient essential appropriate to some of l_0_, $l_{1}$, $L_{0}$ and $L_{1}$ set. Discover the group number of the coefficient of D for each curvelet coefficient. If the group is not identical to G_i_ then find the group number of subsequent coefficients of curvelet. Or else, patient information is inserted into D by using the following equations.

(9) $$\{\begin{array}{c} D\prime =D-\frac{1}{A}*radi_{12}\;\mathrm{if}\;(l_{0}\geq D\geq L_{1})\\ D\prime =D-\frac{1}{A}*radi_{11}\;\mathrm{if}\;(l_{1}\geq D\geq L_{0})\\ D\prime =D\;\mathrm{Otherwise} \end{array}$$

## 4. Data Extraction

A reversible blind extraction process is presented in this section that mined an embedded watermark from the ECG signals. In this work, the original watermark is not required to extract of the patient’s information. The ECG embedded with patient’s information i.e., watermarked ECG is firstly converted to 2D-ECG signal as the procedure discussed in Section 3.1. To convert time-domain image into frequency domain curvelet transform was applied on 2-D watermarked ECG image and then select course level of curvelet transform. The selected scale’s coefficients are classified into clusters as discussed in Section 3.4 above. To extract the embedded watermark, use the embedding clusters, i.e., R_(X−1)_ to R_−(X−1)._ Then for each selected cluster calculate $l_{0}\left(q\right),{\;l}_{1}\left(q\right),{\;L}_{0}\left(q\right),{\;L}_{1}\left(q\right)$ by using ${\mathrm{Radi}}_{i1}$, ${\mathrm{Radi}}_{i2}$ as discussed in Equations (5)–(7). Then for each selected cluster calculate extract bits of the watermark by using the below equation.

(10) $$\{\begin{array}{c} w=1\;\mathrm{if}\;\left(L_{0}\leq D\leq L_{1}\right)\wedge \left(A\%\;2==1\right)\\ w=1\;\mathrm{if}\;\left((l_{0}\leq D\leq l_{1}\right)\wedge \left(A\%\;2==1\right)\\ w=1\;\mathrm{Otherwise} \end{array}$$

## 5. Pseudo Code

### 5.1. At Transmission: Embedding

**Input:** patient information as text data and 1D-ECG signal.

**Output:** watermarked ECG image.

The pseudo-code for embedding patient information into the curvelet domain using clustering is given below:

1. Read patient information, convert this text information into image. To process, the patient information or to make the watermark more imperceptible this patient information image, i.e., in RGB format converts it into a 2-D grayscale image;
2. Read ECG signal as the original signal and detect the QRS wave in this ECG signal to resist the diagnose detection;
3. For detection of QRS wave Pan–Tompkins function was used. Here the parameters of this Pan–Tompkins function are ECG signal, frequency and number;
  - [qrs_amp_raw, qrs_i_raw,delay,final] = pan_tompkin(Orig_Sig,128,1)
4. To process the ECG signal in time domain first, convert this 1D-ECG signal into a 2D-ECG image;
5. Second, to adapt the environment of frequency domain this 2D-ECG image is converted into the transformed domain by using curvelet transform;
6. After this from this frequency domain, the embedding domain is selected. As discussed coarse-level coefficients maintain most of the energy of image to give more invisibility. The coarse-level coefficients were designated as the embedding domain;
7. Now categorized the selected embedding domain into different clusters as discussed in Section 3.4 above;
  - Find the dissimilarity matrix;
  - Calculate clustering;
  - Making the clusters by obtaining the values of centers and the radius of the cluster;
8. Then from these clusters set, select the cluster to hide the watermark. Let, it be group R_i_ to R_j_. Change name the particular cluster G_1_, G_2_ … G_k_, where k = 2(I − j) + 2. For each cluster G_i_, the radii of the groups are ${\mathrm{radi}}_{i1}=c_{i}-\max\left(G_{i}\right)$, ${\mathrm{radi}}_{i2}=c_{i}-min(G_{i}$) as discussed in Section 3.5 above;
9. Now, using Equations (5)–(8), to calculate the values of l_0_, $l_{1}$,${\;L}_{0}$ and $L_{1}$ set;
10. For each coefficient, if the coefficient belongs to the selected cluster G_i_ the watermark image bit is inserted by modifying the coefficient value by using Equation (9). Otherwise find the group number of subsequent coefficients of curvelet;
11. With these updated coefficients values the ECG image is converted into the time-domain by applying the inverse curvelet transform. Then change the 2D-ECG image into 1D-ECG signal. The resulted signal is a watermarked signal.

### 5.2. At Receiving: Reversible Blind Extraction

1. Read watermarked 1D-ECG signals that embedded with the patient’s information;
2. This watermarked ECG signals is altered into a 2D-ECG image as the procedure discussed in Section 3.1;
3. To convert time-domain image into frequency domain curvelet transform was applied on 2-D watermarked ECG image;
4. Then form this frequency domain the course level was selected. The selected scale’s coefficients are classified into clusters as discussed in Section 3.4 above;
5. The selected clusters used in the embedding procedure numbered and treated as the key in the extraction process;
6. Then for each selected cluster calculate $l_{0}\left(q\right),{\;l}_{1}\left(q\right),{\;L}_{0}\left(q\right),{\;L}_{1}\left(q\right)$ by using ${\mathrm{Radi}}_{i1}$, ${\mathrm{Radi}}_{i2}$ as discussed in Equations (5)–(7);
7. Then for each selected cluster extract bits of the watermark by using Equation (10). Now convert these bits into the 2-D image to get the extracted watermark image, i.e., text data of patient information in the form of an image.

## 6. Experimental Results

The demonstration of the presented work was assessed using parameters such as imperceptibility, data loss and the effect of ability to diagnose. Peak signal to noise ratio (PSNR), normalized correlation (NC), PRD and KL metrics were used to measure the imperceptibility and effect of ability to diagnose. Data loss can be estimated using MSE, bit error rate (BER) and SSIM [27]. In this, $X_{c}$ was the host ECG image and $X_{w}$ was watermarked ECG, M × N was the size of image M = no. of rows and N = number of columns.

**A.**  **Peak signal to noise ratio (PSNR):** The PSNR is usually expressed in terms of the logarithmic decibel scale. The following expression was used to calculate PSNR between two images.
  (11) $$PSNR=20\log_{10}\left(\frac{\max\left(X_{c}\right)}{\sqrt{\frac{1}{M*N}}\sum_{i=1}^{N}{\left[X_{c}-X_{w}\right]}^{2}}\right)$$
**B.**  **Normalized correlation (NC):**
  (12) $$NC=\frac{\sum_{i,j}\left(X_{c}{*X}_{w}\right)}{\sum_{i,j}X_{c}{}^{2}}$$
**C.**  **Bit error rate (BER):**
  (13) $$BER\left(w,w^{*}\right)=\frac{\sum_{i,j}X_{c}⊕X_{w}}{\left(M*N\right)}$$
**D.**  **Percentage residual difference (PRD):**
  (14) $$\mathrm{PRD}\%=\sqrt{\left(\frac{\sum_{i=1}^{N}{\left(X_{c}-X_{w}\right)}^{2}}{\sum_{i=1}^{N}{\left(X_{c}\right)}^{2}}\right)}*100$$
**E.**  **Kullback–Leibler divergence (KL):**
  (15) $$D\left(p_{c},p_{w}\right)=\int^{\;}p_{c}\left(x\right)\log\frac{p_{w}\left(x\right)}{p_{c}\left(x\right)}\mathrm{dx}$$
  where D = KL divergence, $p_{w}$ = probability x event in the watermarked, $p_{c}$= probability of the host signal

All other metrics are access information in time domain only KL divergence calculated in frequency domain it will advantageous when the signals are frequency domain [28].

**F.**  **Structure similarity index measure (SSIM):** PSNR was the traditional error summation approach for evaluating the similarity, but PSNR matric only shows the difference between the image intensity, it does not relate with the quality [29,30]. Hence, a new approach was developed by Wang [29] to measure the comparison among the host and disturbing image.
  (16) $$\mathrm{SSIM}=\frac{\left(2\;\partial_{i}\partial_{j}+\omega_{1}\right)\left(2\beta_{j}+\omega_{2}\right)}{\left(\partial_{i}^{2}+\partial_{j}^{2}+c_{1}\right)\left(\beta_{i}^{2}+\beta_{j}^{2}+\omega_{2}\right)}$$
  where $\partial_{i}=\frac{1}{M}\overset{M}{\underset{i=1}{\sum }}i_{i}$, $\partial_{j}=\frac{1}{M}\overset{M}{\underset{i=1}{\sum }}{\hat{i}}_{i}$, $\beta_{i}=\sqrt{\left(\frac{1}{M}\overset{M}{\underset{i}{\sum }}{\left(i_{i}-\partial_{\mathrm{xi}}\right)}^{2}\right)}$, $\beta_{j}=\sqrt{\left(\frac{1}{M}\overset{M}{\underset{i}{\sum }}{\left({\hat{i}}_{i}-\partial_{i}\right)}^{2}\right)}$, $\omega_{1\;}={\left(t_{1}P\right)}^{2}$,$\omega_{2}={\left(t_{2}P\right)}^{2}.\;t$_1_ = 0.01, t_2_ = 0.03, P = 255 (maximum value of host ECG).

**Figure 4:**
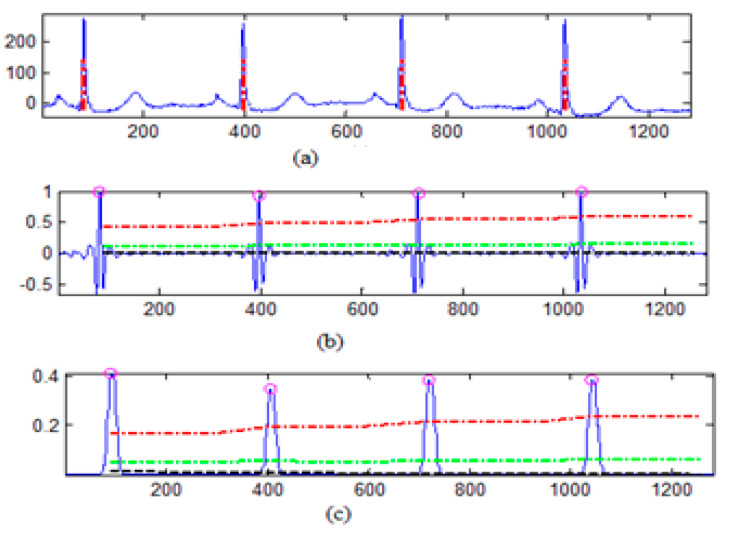
QRS detection (**a**) pulse train of the QRS on ECG signal (**b**) QRS on filtered signal (**c**) QRS on signal and noise level (black), signal level (red) and adaptive threshold (green).

**Figure 5:**
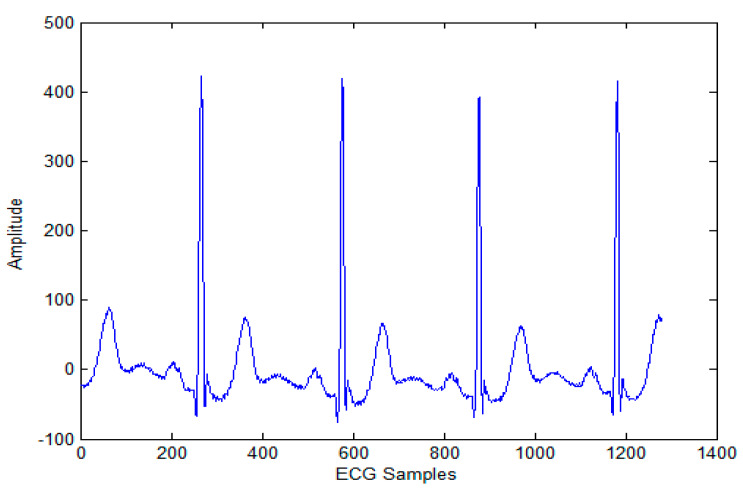
Watermarked ECG.

The MIT-BIH arrhythmia database was used to collect the ECG signals [19] sampled at 128 Hz. The original 1D-ECG signal is shown in Figure 1 whereas the 2D-ECG signal is shown in Figure 2. The QRS complex attributes form the original ECG are shown in Figure 4 and Figure 5 show the resulting image of the proposed embedding procedure, i.e., watermarked ECG signal. The visual quality of the original 1D-ECG signal is the same as watermarked 1D-ECG. The experimental result also concluded that the watermarked image preserved the attributes QRS complex. The original and information embedded ECG, i.e., watermarked ECG both show a higher level of similarity.

The performance of the proposed technique is analyzed by different ECG signals. The same watermark as shown in Figure 3 is embedded into the curvelet coefficients of the ECG signal. The selected clusters are 0–7, so the size of the watermark that embedded is 128 × 128. Table 1 shows the performance of the proposed technique with different metrics. The Stego ECG does not affect the ability to diagnose and as well as the signal disturbance is minimum. The PSNR is greater than 65 for all ECG signals to verify the quality of the original and Stego ECG is very similar. The values of NC, BER, MSE and SSIM show the comparative analysis of both Stego as original ECG. Values of KL and PRD shows that the signal contains minimum disturbance.

The projected watermarked ECG has confirmed besides the dissimilar size of information and the cluster size the experimental results are accessible in Table 2. The visual quality of the original signal may be degraded by the size of embedded information. The discussed algorithm does not reduce the visual quality it delivers a good imperceptibility to the hidden information. As well as the discussed algorithm also takes care the embedded information will not modify the QRS attribute as well as not affect the ability to diagnose the disease. The experimental results shown in Table 2 verified the performance of the technique. The technique was verified by embedding different sizes of the watermark in different cluster sizes. In Table 2 the performance measures metrices KL, MSE, PRD and BER confirm the superiority of the discussed technique. Table 2 verified the similarity of the original ECG signal and watermarked ECG signal with reference to PSNR (which was above 65). Further, the values of NC and SSIM are one or near to one.

**Figure 6:**
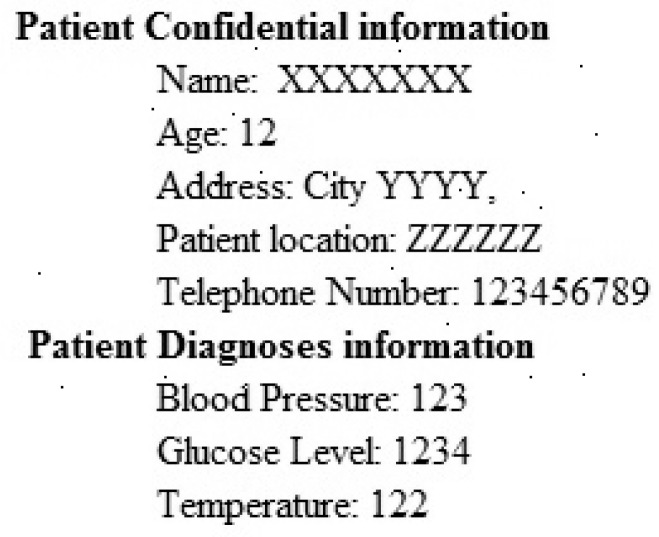
Extracted patient information.

The quality of extracted information was furthermore compared with the original information. Figure 6 demonstrates the extracted patient information and whereas the performance of the extracted information was being compared by the embedded one. The values of PSNR, i.e., 65.31, NC = 1, SSIM = 0.991 and MSE = 1.864 × 10^4^ verified the quality of extracted information was similar to embedded one.

Table 3 shows the quality of the extracted patient’s information. The text information of patients was converted into an image and embedded into the curvelet transform by using clustering. Table 3 shows the visual quality of the patient’s information. The cluster was 0–7 and the size of the embedded information was 128 × 128. The quality of the extracted information was analyzed by PSNR, NC and SSIM metrics. The values of PSNR verify the visual quality of the extracted information was above 64. The values of NC are one for all ECG signals and values of SSIM are near to one that shows the similarity between the embedded and extracted images.

The implanted information may be affected by image-processing operations like rotation, filtering, cropping, compression, etc. To demonstrate the superiority against the image-processing operation, the technique also extracted the information from the noised, filtered, rotated, compressed and cropped ECG signals. Table 3 shows the values of the PSNR and MSE after compared the extracted and embedded information from distorted ECG. Table 4 verified the robustness of the technique because the extracted watermark was very similar to the original one.

**Figure 7:**
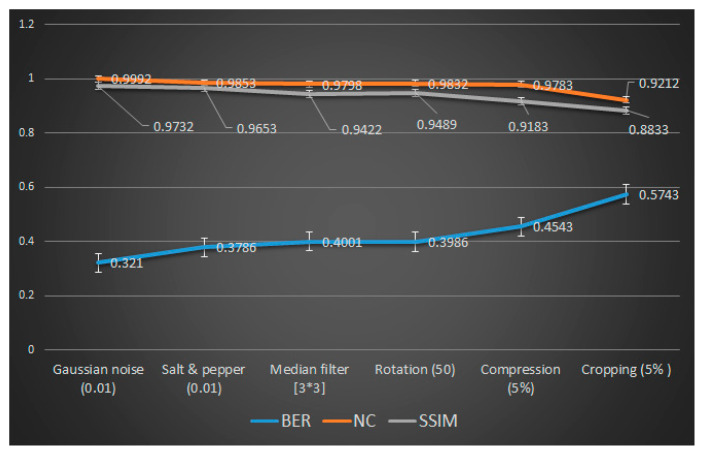
Robustness of proposed techniques corresponding to different attacks.

Figure 7 shows the values of BER, NC and SSIM of extracted patient images and embedded images under different image-processing attacks. The values of BER was less than 0.5 in all cases even the values of NC and SSIM are near to one. The values of BER, SSIM and NS confirm the resistance of the image-processing attacks on the extracted watermark. Therefore, from Table 4 and Figure 7 demonstrate the robustness of the presented technique against image-processing attacks (Y-Axis).

Some authors [14,15,16] have used the curvelet transform for embedding the information into the ECG signals as discussed above. In the adaptive threshold [15] and the quantization approach [16] authors proposed the steganography ECG using curvelet. In their study, they followed the steganography definition to make watermark invisible. Hence, these techniques first emphasized the imperceptibility of embedding information. Further improvement for the robustness of the watermark was still a critical parameter left in the previous work. Second, they used binary data as watermark. Binary data are very fragile because any mirror change may lose the data or change the meaning. High levels of false-positives were detected while using binary data as a watermark.

To highlight the superiority of the proposed technique, HH scale DWT-SVD [12], adaptive threshold [15] and quantization approach [16] some latest ECG-watermarking techniques results were compared. In this technique, the author used clustering and some watermark-strength parameters to make the watermark invisible, as well as robust. The algorithm performance metrics like KL, PRD, MSE, etc., are being compared by existing ECG-watermarking techniques are shown in Table 5. If the 67 × 67 size of information was embedded into the ECG signals the value of PSNR value of the watermarked ECG was 78.0702 dB. Subsequently, the values of PSNR show the resemblance among the host ECG signal and watermarked ECG signal. As the value of PSNR achieved by the quantization approach was 73.75 dB, which can make a base when accompanied by the performance estimation. However, comparing the PSNR values of HH scale DWT-SVD and adaptive threshold techniques are simply 50.44 dB and 60.68 dB, respectively. Coefficients whose values are zero are exploited in the quantization approach. However, the embedding capacity of the quantization approach was very low; in this proposed technique further improvement to insert more watermarks bits with zero values coefficient may be achieved. To locate the watermark, an n × n sequence was used in the adaptive threshold method. In this method, the author also compromised with the embedding capacity. The proposed technique was not restricted in terms of embedding capacity because the technique used the property of clustering to hide the watermark. The collection of coefficients depends on the size of the implanting watermark. Preferably, the coefficients with the smallest values are finest to insert the patient’s information because they offers the best invisibility.

To embed the information the text information was transformed into binary in HH scale DWT-SVD [12], adaptive threshold [15] and quantization approach [16] techniques. The whole information of any patient may be changed if there any false positive detection because the information was inserted into a binary form. However, in the projected technique image embedded into the ECG. The text information was first transformed into the image and this image was inserted by modifying the curvelet coefficients using a clustering approach. The benefit of embedding an image was that false-positive detection will only change the pixel intensity that only degrades the image quality. The process can be automated using deep learning [30]. However, the meaning of the entire embedded information was not changed. The proposed ECG-watermarking technique that offers not only the best invisibility of watermark as well as it also offers good robustness and secures ECG-watermarking.

## 7. Discussion

In this presented technique, the authors embedded patient information as a watermark into ECG signals. As the coarse-level maintains most of the energy of image, this level was chosen to make the watermark more robust. To obtain the coarse-level coefficients, clustering approaches were used. To verify the strength of the cluster, the watermark was embedded into the ECG signal. Even when the watermark was embedded as a text image, some false-positive occurred, that not only degraded the quality of the extracted watermark, but also effected the robustness of the image. Hence, the text must be the embedded as a single unit. The method applies to some biometric techniques like fingerprint, MRI, CT-scan, etc. However, to obtain optimum results, the use of morphologic operators may be different. Moreover, QRS waves are not used in all types of biometrics. Additionally, the work was limited to images only, as it was applicable only for embedding the text watermark. So algorithm with steganography may need further dataset. Today, with everything digital for security reasons, said methodology may be used for better encryption. To enhance the scope of the technique utilization of deep learning, machine learning may be incorporated as a further improvement for automation. In a nutshell, as a medical application, the work can be extended to medical video-watermarking, voice-watermarking and online-content security, etc.

## 8. Conclusions

A curvelet transform-based ECG-watermarking approach using Euclidean and non-similarity distance clustering technique was presented and a 1D-ECG was converted into a 2D-ECG image by preserving the QRS complex. The curvelet transforms 2D-ECG results from a set of coefficients. A Euclidean, non-similarity distance method was incorporated to make the clusters of the coefficients. Patient information was transformed into the image and embedded into the selected cluster coefficients. Keys were utilized to secure patient information from unauthorized access. The performance of the proposed method was evaluated by metrics such as PSNR, NC, PRD, KL, BER, MSE and SSIM. As the size of the patient’s information increase, the quality of the signal decreases, but this does not affect the ability to diagnose disease from the ECG. It was verified by experimental results that the BER was approximately zero, and the NC was zero by a two-fold increase in the patient information. Patient information was also secure and robust from any geometric attacks. The proposed technique can be easily implemented in wearable medical devices to transmission of the ECG. Hence, the proposed technique has provided a better way to transfer patient information without affecting the ability to diagnose, and provides a reliable, secure and robust ECG-watermarking technique. However, there is always room for improvement. Deep learning and fuzzy logic may be applied for non-singularity and varying levels of brightness/noise. The automation of the process can be improved at the early stages for even better results.

## Figures and Tables

**Table 1:** Performance of proposed techniques (Peak signal to noise ratio (PSNR),Normalized correlation (NC), Kullback–Leibler divergence (KL),Mean Square Error(MSE),Percentage residual difference (PRD),Bit error rate (BER),,Structure similarity index measure (SSIM).

Sr. No.	ECG Signal	PSNR	NC	KL	MSE	PRD	BER	SSIM
1	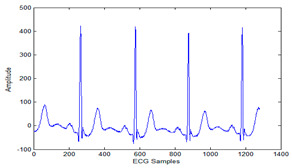	66.825	1.00	0.0012	0.0011	0.0967	0.24	1.000
2	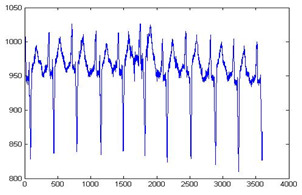	68.342	1.00	0.0009	0.0009	0.0653	0.194	1.00
3	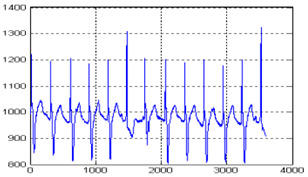	70.043	1.00	0.0002	0.00043	0.021	0.142	1.00
4	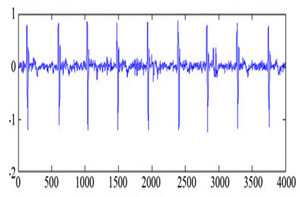	69.8562	1.00	0.0005	0.0007	0.043	0.174	1.00

**Table 2:** Performance of proposed techniques with different watermark sizes and clusters.

Cluster	0–1	0, 1	0, 1, 2	0, 1, 2, 3	0–7
**Watermark Size**	32 × 32	64 × 64	80 × 80	100 × 100	128 × 128
**PSNR**	84.5131	79.7489	83.6277	74.2855	66.8254
**NC**	1	1	1	1	1
**KL**	2.00 × 10^−5^	2.15 × 10^−5^	0.0001	0.0007	0.0012
**MSE**	7.67 × 10^−5^	7.26 × 10^−5^	0.0001	0.0005	0.0011
**PRD**	0.0421	0.0545	0.0494	0.0674	0.0967
**BER**	0	0	0	0.011	0.214
**SSIM**	1	1	1	1	1

**Table 3:** Quality of extracted patient’s information.

Sr. No.	ECG Signal	Extracted Watermark	PSNR	NC	SSIM
1	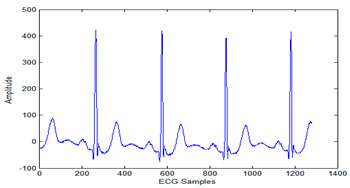	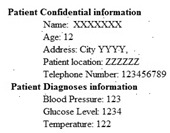	65.31	1	0.911
2	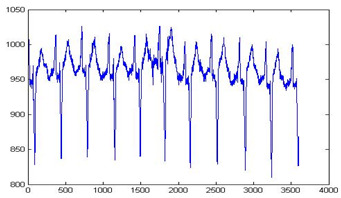	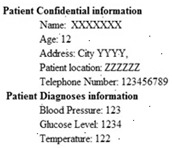	65.89	1	0.934
3	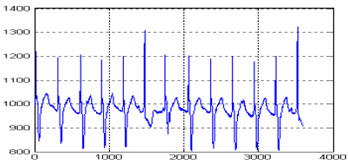	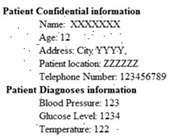	64.983	1	0.974
4	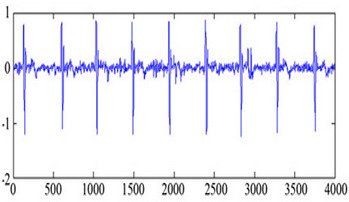	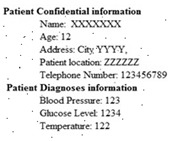	64.9653	1	0.9832

**Table 4:** Quality of extracted watermark after different image-processing operations.

Operations	Gaussian Noise (0.01)	Salt & Pepper (0.01)	Rotation (5°)	Compression (5%)	Median Filter (3 × 3)	Cropping (5%)
**PSNR**	43.6168	42.5698	41.49	38.2123	40.2738	32.1112
**MSE**	2.10 × 10^−3^	6.10 × 10^−2^	0.0421	0.0761	0.0213	0.1821
**BER**	0.321	0.3786	0.4001	03,986	0.4543	0.5743
**NC**	0.9992	0.9853	0.9798	0.9832	0.9783	0.9212
**SSIM**	0.9732	0.9653	0.9422	0.9489	0.9183	0.8833

**Table 5:** Comparison of proposed technique with existing ECG techniques.

Performance Metric	Watermark Size	PSNR	KL	MSE	BER	PRD
**HH Scale DWT-SVD** [12]	67 × 67 (4489 bits)	50.44	0.15	0	0	0.59
**Adaptive Threshold Method** [15]	251 bytes (2008 bits)	60.68	0.0027	0.05	0	0.0018
**QuantizationApproach** [16]	251 bytes (2008 bits)	73.75	0.00023	0.002	0.04	0.04
**Proposed Technique**	67 × 67 (4489 bits)	78.0702	0.0000455	3.38 × 10^−4^	0	0.105
